# Infective Endocarditis by *Capnocytophaga* Species—A Narrative Review

**DOI:** 10.3390/medicina60030382

**Published:** 2024-02-24

**Authors:** Despoina Spentzouri, Stella Baliou, Petros Ioannou

**Affiliations:** 1 Internal Medicine, University Hospital of Heraklion, 71110 Heraklion, Greece; 2 School of Medicine, University of Crete, 71003 Heraklion, Greece

**Keywords:** infective endocarditis, *Capnocytophaga*, *Capnocytophaga canimorsus*

## Abstract

Bacteria belonging to the genus *Capnocytophaga* are thin, capnophilic, Gram-negative bacilli with tapered ends that include nine species that are isolated from the mouth of humans and animals and, from a phylogenetical perspective, they belong to the family *Flavobacteriaceae*. Two more species, namely *C. endodontalis* and *C. stomatis* have been recovered from a periapical abscess and human and animal infections, respectively. *Capnocytophaga* spp. can cause serious and potentially life-threatening infections in humans, such as bacteremia and meningitis, most commonly in the context of penetrating trauma as a result of contact with animals, especially after animal bites. Other invasive diseases such as osteomyelitis, septic arthritis, and infective endocarditis (IE) may also occur more rarely. The aim of this study was to review all previously described cases of IE by *Capnocytophaga* spp. and provide information about the epidemiology, microbiology, antimicrobial susceptibility, clinical characteristics, treatment, and outcomes of this infection. A narrative review based on a search in PubMed, the Cochrane Library, and Scopus was performed. Studies published until 11 September 2023 providing relevant data for IE caused by *Capnocytophaga* spp. in humans were included. A total of 31 studies containing data from 31 patients were included. A history of dog bites was present in 10 out of 26 patients (38.5%). A prosthetic valve was present in 3 patients (9.7%). The most commonly infected valve was the aortic valve, followed by the tricuspid valve. Fever, embolic phenomena, paravalvular abscess, and sepsis were the most common clinical presentations. Beta-lactams and aminoglycosides were the antimicrobials most commonly used. Surgery was performed in 20 patients (64.5%). Overall mortality reached 16.1%.

## 1. Introduction

Infective Endocarditis (IE) is an infection involving the heart valves or any intracardiac prosthetic material such as defibrillators, pacemakers, prosthetic heart valves, or left ventricular assist devices, and is associated with significant morbidity and mortality [1,2]. The microorganisms that are more commonly identified as causes of IE classically include aerobic Gram-positive bacteria, such as staphylococci, streptococci, and enterococci. Several studies have identified these pathogens as the cause of up to 75% of IE cases [3,4]. IE by Gram-negative bacteria is rarely diagnosed, and in most of these cases, members of the Enterobacterales order, such as *Escherichia coli* or *Klebsiella* spp., are identified [3,4]. IE by *Capnocytophaga* spp. has been mainly described through case reports in the literature. Thus, the exact characteristics of IE by these bacteria have yet to be adequately described [5,6].

Bacteria belonging to the genus *Capnocytophaga* are thin, capnophilic, Gram-negative bacilli with tapered ends that include nine species, namely *C. canimorsus*, *C. canis*, *C. sputigena*, *C. gingivalis*, *C. ochracea*, *C. haemolytica*, *C. leadbetteri*, *C. granulosa*, and *C. cynodegmi* that are isolated from the mouth of humans and animals and, from a phylogenetical perspective, they belong to the family *Flavobacteriaceae* [7,8,9,10]. Two more species, namely *C. endodontalis* and *C. stomatis*, have been recovered from a periapical abscess and human and animal infections, respectively [7,11]. *Capnocytophaga* spp. are members of the oral microbiome and have been associated with periodontal disease, oropharyngeal mucositis, and gingivitis [12]. The most common infections caused by *Capnocytophaga* spp. are bacteremia, central nervous system infections, ocular infections, infections associated with pregnancy, and osteoarticular infections [9]. There seems to be a closer association of human infection with contact with dogs rather than with other animals [13,14]. Bacteremia with sepsis is the most common and serious clinical syndrome associated with these microorganisms. Sepsis is most commonly seen in patients with hematological malignancy, and its onset mostly follows the onset of severe neutropenia after chemotherapy [15,16,17]. Overall mortality in patients with bacteremia by *Capnocytophaga* spp. may be up to 42%, with recent studies reporting quite lower rates that may be close to 0% [15,16,18,19]. Central nervous system infections are more commonly seen in immunocompetent individuals after recent dental work, while ocular infections may include keratitis, blepharoconjunctivitis, and endophthalmitis and are more commonly seen in patients who are older or immunosuppressed [9]. Infections in pregnancy may include chorioamnionitis, which is associated with an increased risk of perinatal complications such as preterm birth, fetal death, or perinatal disease in the offspring [9,20]. Other less frequent infections noted include osteomyelitis, peritonitis, aspiration pneumonia, urinary tract infections, and IE [5,6,9,21,22,23]. People with immunosuppression, such as asplenia or neutropenia, as well as other conditions, such as alcoholism, are at an increased risk of developing life-threatening infections from *Capnocytophaga* spp., and more specifically, from *C. canimorsus* (previously known as dysgonic fermenter-2) which is the most commonly isolated species in infected humans [24,25]. *Capnocytophaga* infections may be on the rise due to the increased number of pet owners, the increased number of occupational exposure to animals, and the increasing number of immunocompromised individuals [9].

The pathogenicity of *C. canimorsus* includes several different mechanisms. For example, *C. canimorsus* may prohibit innate immune responses secondary to evasion of phagocytosis by the host’s macrophages and resistance to complement-mediated bacterial cell lysis. Moreover, reduced reactivity of pattern-recognition receptors such as Toll-like receptor 4 may lead to inappropriate immune activation [26]. Additionally, the production of sialidase and cell surface lipoproteins may deglycosylate the host’s glycoproteins, such as immunoglobulins, thus enhancing bacterial persistence [9,27]. However, *C. canimorsus* strains are not all equally pathogenic for humans [28]. For example, even though there are nine serotypes of *C. carnimorsus*, three of them are responsible for about 90% of infections in humans, namely A, B, and C. Importantly, these serotypes are found in less than 10% of dogs’ oral microbiome [29]. This may be associated with the relatively low likelihood of *C. carnimorsus* infections in humans after a dog bite [9].

Given the rarity of IE caused by *Capnocytophaga* species, evidence regarding this condition in the literature is mainly based on case reports with or without a literature review [5,6]. The aim of this study was to review all previously described cases of IE by *Capnocytophaga* species and provide information about the epidemiology, microbiology, clinical characteristics, treatment, and outcomes of these infections.

## 2. Methods

This narrative review extracted and collected data regarding *Capnocytophaga* spp. IE cases in humans. The primary aim of the present study was to provide information regarding the mortality and the epidemiology of these infections. Presenting data on (a) the exact site of infection, (b) the patients’ clinical characteristics, (c) the microbiology of the infection, and (d) their treatment were among the secondary outcomes of this study. For this review, PubMed/Medline, Cochrane Library, and Scopus databases were searched for eligible articles reporting “*Capnocytophaga* AND endocarditis” until 19 November 2023. Inclusion criteria for this review included (a) studies providing original data, such as case reports, case series, and retrospective and prospective studies that provided data at least about epidemiology and outcomes on IE by *Capnocytophaga* species in humans. Articles that were not in English were excluded. Letters to the editor, reviews, and systematic reviews were excluded since they could not provide any original information in the synthesis of this review. Articles without access to original data and studies referring to animal reports were excluded from further analysis. Furthermore, studies that did not have sufficient data on patients’ mortality and epidemiology were also excluded from further analysis. The remaining articles’ references were also searched to assess potential studies following the snowball procedure.

The extracted data included year of publication, study type, and country; patients’ demographics (age and gender); patients’ relevant medical history (previous cardiac surgery or cardiac valve replacement, time after valve replacement, dog or other animal bite); infection and relevant microbiology (infection site, microorganism identification, complications, and embolic phenomena); treatment administered; surgical management (if any), and outcomes (i.e., cure or death). The association of mortality with the infection and causal microbiology was reported according to the study authors. In each case, the diagnosis of IE was confirmed by the current study’s investigators, based on the data provided by the authors in each study and the modified 2023 Duke-ISCVID criteria if the diagnosis was at least possible (at least 1 major and 1 minor criterion or at least 3 minor criteria) or if adequate pathological data justified a diagnosis of IE [30].

Data are presented as numbers (%) for categorical variables and median (interquartile range, IQR) for continuous variables. Continuous variables were compared using the Mann–Whitney U-test for non-normally distributed variables or the t-test for normally distributed variables. All tests were two-tailed, and a *p*-value equal to or lower than 0.05 was considered significant. A univariate linear regression analysis was conducted to identify factors associated with all-cause mortality of patients. More specifically, univariate logistic regression was performed to identify any association between gender, age, presence of prosthetic cardiac valve, bad teeth hygiene or recent dental work, history of previous episode of IE, history of rheumatic heart disease, dog or other animal bites, location of the infection (mitral, aortic, tricuspid, pulmonary, or IE at multiple valves), presence of fever, embolic phenomena, sepsis, heart failure, antimicrobial treatment and surgical management, with all-cause mortality. Statistics were calculated with GraphPad Prism 6.0 (GraphPad Software, Inc., San Diego, CA, USA).

## 3. Results

### 3.1. Included Studies’ Characteristics

**Figure 1:**
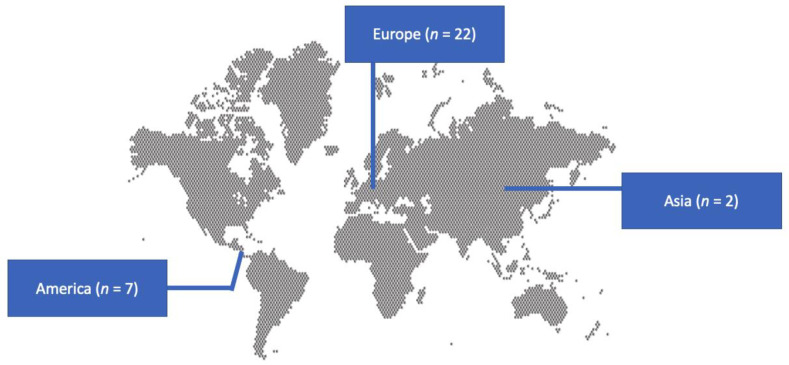
Geographical distribution of studies reporting infective endocarditis by *Capnocytophaga* species worldwide.

A total of 161 articles from PubMed and Scopus were screened. Finally, 31 met the present study’s inclusion criteria [5,6,31,32,33,34,35,36,37,38,39,40,41,42,43,44,45,46,47,48,49,50,51,52,53,54,55,56,57,58,59]. These 31 studies involved 31 patients in total. Among those studies, 22 were conducted in Europe, 7 in North and South America, and 2 in Asia. There were 31 case reports. Figure 1 shows the geographical distribution of *Capnocytophaga* species IE cases worldwide.

### 3.2. Epidemiology of IE by Capnocytophaga Species

**Figure 2:**
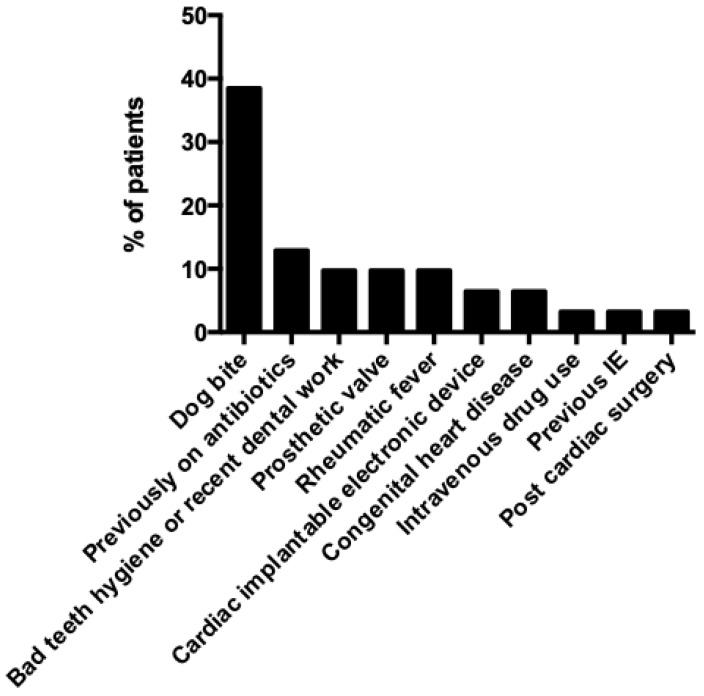
Epidemiology of patients with infective endocarditis by *Capnocytophaga* species. IE: infective endocarditis.

The age of patients with IE by *Capnocytophaga* species ranged from 30 to 76 years; the median age was 56 years, and 74.2% (23 out of 31 patients) were male. Regarding predisposing factors, 38.5% (10 out of 26 patients) had a history of dog bites, 11.1% (3 patients) had a history of a dog licking a wound or other source of microorganism entry), 3.7% (1 out of 27) had a history of another animal bite (lion), 12.9% (4 out of 31) had received antimicrobials during the three months preceding the infection, 9.7% (3 out of 31) had prosthetic cardiac valve, 9.7% (3 out of 31) had history of rheumatic fever, 9.7% (3 out of 31) had had recent dental work or bad oral and teeth hygiene, 6.5% (2 out of 31) had congenital heart disease, 3.2% (1 out of 31) had a cardiac surgery during the three months preceding the infection, 3.2% (1 out of 31) had a previous episode of IE, while 3.2% (1 out of 31) had history of intravenous drug use (IVDU). The patients’ characteristics and infection outcomes can be seen in Table 1. Epidemiology of patients can also be seen in Figure 2.

### 3.3. Microbiology, Antimicrobial Resistance, and Diagnosis of IE by Capnocytophaga Species

**Figure 3:**
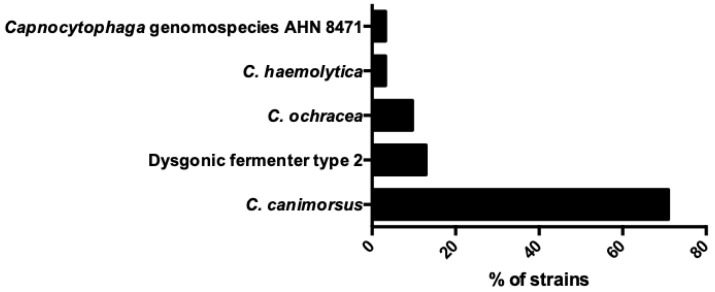
Microbiology of infective endocarditis by *Capnocytophaga* species.

IE by *Capnocytophaga* species was polymicrobial in 3.2% (one patient), with blood cultures being positive both for *Capnocytophaga* genomospecies AHN 8471 and *Streptococcus mitis*. The isolated species from the 31 patients with IE were *C. canimorsus* in 71% (22 patients), dysgonic fermenter type 2 in 12.9% (4 patients), *C. ochracea* in 9.7% (3 patients), *C. haemolytica* in 3.2% (1 patient), and *Capnocytophaga* genomospecies AHN 8471 3.2% (1 patient). Figure 3 shows the microbiology of IE by *Capnocytophaga* species. The method for microorganism identification was not mentioned in 38.7% (12 out of 31 patients). The most common identification method was 16s rRNA PCR in 35.5% (11 patients), the matrix-assisted laser desorption/ionization time-of-flight mass spectrometry (MALDI-TOF MS) in 16.1% (5 patients), and classic microbiology methods in 16.1% (5 patients). Antimicrobial resistance to aminoglycosides was 66.7% (8 out of 12 strains with available data), to penicillin was 5.6% (1 out of 18 strains), to ampicillin was 0% (0 out of 11 strains), and to cephalosporins was 0% (0 out of 15 strains).

Diagnosis of IE by *Capnocytophaga* species was facilitated by transthoracic echocardiography in 66.7% (20 out of 30 patients), transesophageal echocardiography in 23.3% (7 patients), valve culture in 16.1% (5 out of 31 patients), and by autopsy in 6.5% (2 patients).

### 3.4. Clinical Characteristics of IE by Capnocytophaga Species

IE by *Capnocytophaga* species affected the aortic valve in 51.7% (15 out of 29 patients with available data), the tricuspid valve in 34.5% (10 patients), the mitral valve in 20.7% (6 patients), and a cardiac implanted electronic device (CIED) in 3.2% (1 patient). Multiple valves were infected in 10.3% (3 patients).

**Figure 4:**
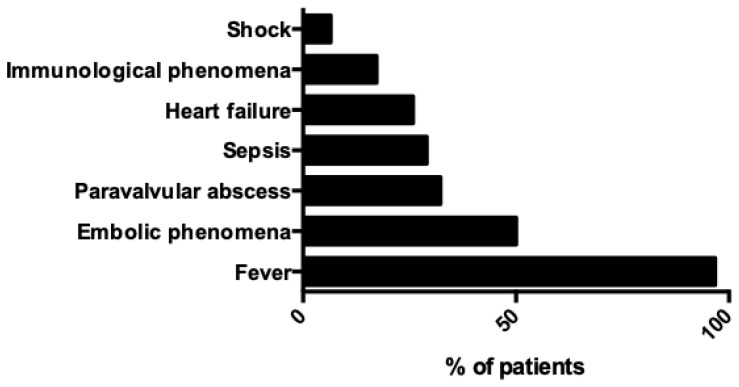
Clinical characteristics of patients with infective endocarditis by *Capnocytophaga* species.

The most common clinical presentation included fever in 96.8% (30 out of 31 patients), embolic phenomena in 50% (15 out of 30 patients), paravalvular abscess in 32.3% (10 out of 31 patients), sepsis in 29% (9 patients), heart failure in 25.8% (8 out of 31 patients), immunological phenomena in 17.2% (5 out of 29 patients) shock in 6.5% (2 out of 31 patients). Figure 4 shows the clinical characteristics of patients with IE by *Capnocytophaga* species.

### 3.5. Treatment and Outcomes of IE by Capnocytophaga Species

**Figure 5:**
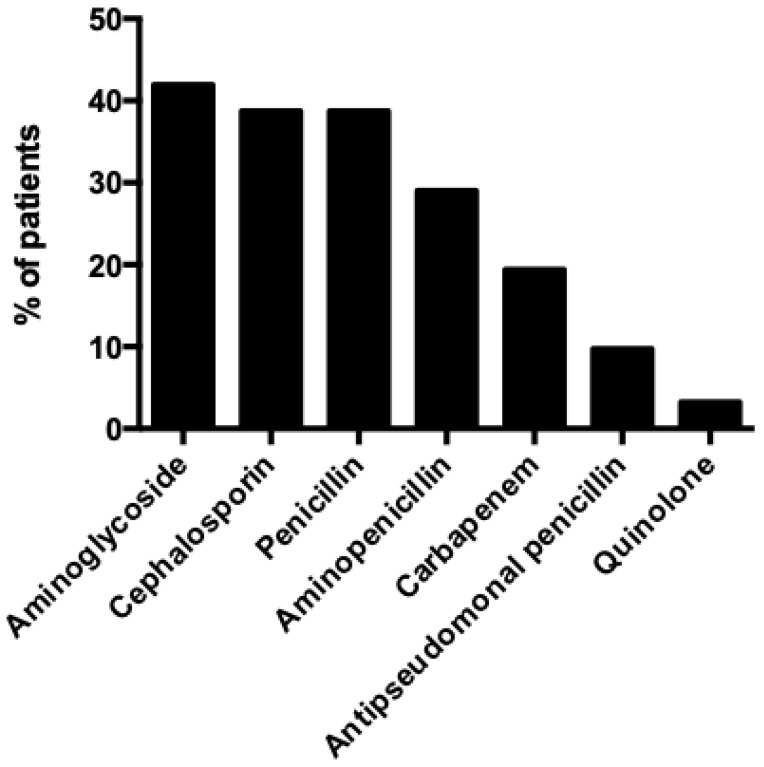
Antimicrobial treatment of patients with infective endocarditis by *Capnocytophaga* species.

Treatment of patients with IE by *Capnocytophaga* species is summarized in Table 1, shown in Figure 5, and described in detail in Table 2. The median treatment among survivors was 6 weeks. The antimicrobials that were used more commonly were aminoglycosides and beta-lactams, and more specifically, penicillin in 38.7% (12 out of 31 patients), cephalosporins in 38.7% (12 patients), aminopenicillins in 29% (9 patients), carbapenems in 19.4% (6 patients), and antipseudomonal penicillins in 9.7% (3 patients). Surgical management, along with antimicrobial treatment, was performed in 64.5% (20 patients). Overall mortality was 16.1% (5 patients), while only in 6.5% (2 patients) the death was directly attributed to the IE episode.

### 3.6. Comparison of Patients with IE by Capnocytophaga Species Who Died with Those Who Survived

Table 1 shows a comparison of patients with IE by *Capnocytophaga* species who died with those who survived. Even though the low number of patients precluded adequate statistical power to draw solid conclusions, patients who died were more likely to have had embolic phenomena in their clinical presentation.

### 3.7. Statistical Analysis of IE by Capnocytophaga Species

In the univariate regression analysis, among the different parameters tested, the diagnosis of IE by *C. canimorsus* was negatively associated with overall mortality (*p* = 0.0049), while the development of embolic phenomena and the treatment with a cephalosporin was positively associated with overall mortality (*p* = 0.0132, and *p* = 0.0395, respectively). Due to the relatively small number of events and the many parameters tested, the conduction of a multivariate logistic regression was not considered to be safe for the extraction of reliable data.

## 4. Discussion

This study presented the epidemiologic and clinical characteristics of patients diagnosed with IE by *Capnocytophaga* species. The most commonly infected valve was the aortic valve, followed by the tricuspid valve. The most common clinical presentation included fever, embolic phenomena, paravalvular abscess, and sepsis. Beta-lactams and aminoglycosides were the antibiotics most commonly used, with penicillin and cephalosporins being the most frequent among the beta-lactams. Overall mortality was 16.1%.

The most commonly identified species in the case of IE in the general population are Gram-positive bacteria, and more specifically, streptococci, staphylococci, and enterococci [3,4]. Blood cultures remain the main method for pathogen identification of the causative pathogen [60]. In all cases where IE is suspected, the receipt of blood cultures is indicated, ideally with the receipt of at least three sets of aerobic and anaerobic bottles before initiating antimicrobial treatment [60]. If a blood culture turns positive, the Gram stain will help discriminate between Gram-negative and Gram-positive bacteria, while further tests evaluating the shape and the biochemical profile of the bacteria will allow further discrimination between different species [61]. Newer molecular techniques such as the matrix-assisted laser desorption/ionization time-of-flight mass spectrometry (MALDI-TOF MS) and the 16s rRNA gene sequencing allow timely pathogen identification with extremely high specificity even in cases where morphology and biochemical tests yield inconclusive results [62].

The patients diagnosed with IE due to *Capnocytophaga* spp. in the current study had a median age of 56 years, which was lower compared to the age of patients with IE in cohorts due to other microorganisms, where the mean age is about 70 years [3,4,63]. A clear male predominance was noted herein, as is also the case in patients with IE due to other bacteria [3,63]. Importantly, 38.5% of patients with IE by *Capnocytophaga* spp. had a history of a dog bite, and 3.7% had a history of a bite from another animal (lion). This is in line with the literature that shows a very close association of infection by these pathogens with the oral cavity of animals and, more specifically, of dogs [14,24,64]. In the present study, 9.7% of patients had a prosthetic valve, while in other studies of patients with IE, that rate was up to 50% [3,4,63]. About 3% of patients in the present study had a previous episode of IE, while the rate of patients with a history of rheumatic fever was 10%. Both rates are close to those noted in other studies of patients with IE in the general population [4,63]. Intravenous drug use was noted in about 3% of the patients in this study, which is close to the range in other studies with patients with IE, being 4% to 9.2% [3,4,63]. Congenital heart disease was noted in 6.5% in the current study, a rate similar to the one noted in another study that reported characteristics of patients with IE [4].

The most commonly infected valve in the current study was the aortic in 51.7%. This complies with some other studies of patients with IE; the aortic valve was the most commonly infected [3,63]. However, herein, the tricuspid valve was the second most commonly infected in 20.7% of patients, while in other studies, the mitral valve is the second most commonly infected [3,63]. The reason for this event is unclear. Classically, IVDU is associated with tricuspid valve endocarditis [65]. The most commonly identified pathogens in the case of right-sided IE are *S. aureus*, which may be the cause in up to 70% of cases, streptococci and enterococci [66,67,68,69]. Gram-negative pathogens are estimated to be the cause of up to 5% of cases of right-sided IE [70]. The increased frequency of tricuspid valve involvement in *Capnocytophaga* spp. IE may warrant further investigation in future studies.

Regarding clinical presentation, the most common symptom of IE in these patients was fever, which was evident in 96.8%, while sepsis was noted in 29%, and 6.5% developed shock. In other studies, fever was noted in 84% [4], and shock was diagnosed in 9% of patients [3]. A diagnosis of heart failure was performed in 25.8% of patients, a rate that is slightly lower than the one noted in other studies that ranged from 33% to 52% [3,63]. Embolic phenomena in the patients presented herein were evident in 50%, which is close to the rate noted in other studies of patients with IE, which was within the range of 15% to 45% [3,4]. Importantly, almost all patients in the studies included in the present review were diagnosed using echocardiography. Notably, since some of these studies report cases from the 1980s, echocardiography’s availability and diagnostic accuracy would differ from those that describe patients with IE diagnosed just a few years ago. Moreover, other technical issues regarding patient variability could also impact the diagnostic accuracy of echocardiography [71,72,73,74].

Beta-lactams were the regimens most commonly used for patients’ treatment. This is reasonable given that the antimicrobial susceptibility of *Capnocytophaga* spp. to penicillin, aminopenicillins, and cephalosporin was very low. This is in line with the literature, where the antimicrobial susceptibility of *Capnocytophaga* spp. to beta-lactams is very high [75]. However, since there are several reports of *Capnocytophaga* strains that produce beta-lactamases, such as in strains that are part of the human oral microbiome, antimicrobial susceptibility testing should always be performed to reduce the possibility of treatment failure [76,77]. In every case, beta-lactams, with or without beta-lactamase inhibitors, remain the first line of treatment for infections by *Capnocytophaga* spp. [78]. Regarding aminoglycosides, antimicrobial resistance was high, as has been previously reported in other studies [79]. Even so, aminoglycoside was used in about 42% of the patients described in the present review in the context of combination antimicrobial therapy. Importantly, when performing studies like the present one, one should consider the prevalent guidelines at that time. This is particularly important in the case of IE by classic pathogens since the use of some antimicrobial drugs, like aminoglycosides, for example, had been traditionally considered valuable in the treatment of IE [80]. However, in recent years, skepticism has grown regarding their use, and they are losing their role in the guidelines [81]. Regarding the treatment of IE by *Capnocytophaga* spp., however, evidence was scarce. Thus, the optimal treatment has largely been unknown until now, and guidelines specifically regarding this disease have not yet been published.

Herein, overall mortality was 16.1%, but only 6.5% of patients died due to the infection. The overall mortality was comparable to that in other studies, where it was within the range of 11–40% [3,4,63].

This study has some limitations that should be mentioned. First, its evidence is derived from a small number of case reports. Thus, the provided evidence may be low. However, given the rarity of this disease, it is unlikely that a large retrospective or prospective study could be performed to provide a large number of patients, even if it had a multicenter design. Second, since the number of patients was relatively small, a multivariate logistic regression analysis for overall mortality could not be performed. Finally, since this study is a narrative review and not a systematic one, the evidence provided is limited.

## 5. Conclusions

This narrative review provides information on IE by *Capnocytophaga* spp. by describing the epidemiology, clinical characteristics, microbiology, antimicrobial susceptibility, antimicrobial therapy, and outcomes of this disease, and provides an informative comparison of these characteristics with the characteristics of IE by other pathogens. Penicillin resistance was very low, and beta-lactams were the antimicrobials most commonly used for treatment. On the other hand, aminoglycoside resistance rates were low; however, these drugs were the second most commonly used for the treatment of this disease in the context of combination treatment with other antimicrobials.

## Figures and Tables

**Table 1:** Patients’ characteristics and infections’ outcome.

Characteristic	All Patients (*n* = 31) *	Survived (*n* = 26)	Died (*n* = 5)	*p*-Value
Age, years, median (IQR)	56 (43–64)	55.5 (42.8–66)	59 (47–64)	0.8061
Male gender, *n* (%)	23 (74.2)	19 (73.1)	4 (80)	1
Predisposing factors				
Dog bite, *n* (%)	10 out of 26 (38.5)	9 out of 21 (42.9)	1 (20)	0.6169
Previously on antibiotics, *n* (%)	4 (12.9)	4 (15.4)	0 (0)	1
Bad teeth hygiene or recent dental work, *n* (%)	3 (9.7)	3 (11.5)	0 (0)	1
Rheumatic fever, *n* (%)	3 (9.7)	3 (11.5)	0 (0)	1
Prosthetic valve, *n* (%)	3 (9.7)	2 (7.7)	1 (20)	0.4216
CIED, *n* (%)	2 (6.5)	2 (7.7)	0 (0)	1
Congenital heart disease, *n* (%)	2 (6.5)	2 (7.7)	0 (0)	1
Post cardiac surgery, *n* (%)	1 (3.2)	1 (3.8)	0 (0)	1
Previous IE, *n* (%)	1 (3.2)	1 (3.8)	0 (0)	1
IVDU, *n* (%)	1 (3.2)	1 (3.8)	0 (0)	1
Method of diagnosis				
Transthoracic echocardiography, *n* (%)	20 out of 30 (66.7)	19 (73.1)	1 out of 4 (25)	0.0952
Transesophageal echocardiography, *n* (%)	7 out of 30 (23.3)	6 (23.1)	1 out of 4 (25)	1
Autopsy, *n* (%)	2 (6.5)	0 (0)	2 (40)	NA
Valve culture, *n* (%)	5 (16.1)	5 (19.2)	0 (0)	0.5601
Valve localization				
Aortic valve, *n* (%)	15 out of 29 (51.7)	13 out of 25 (52)	2 out of 4 (50)	1
Tricuspid valve, *n* (%)	10 out of 29 (34.5)	8 out of 25 (32)	2 out of 4 (50)	0.5920
Mitral valve, *n* (%)	6 out of 29 (20.7)	5 out of 25 (20)	1 out of 4 (25)	1
Multiple valves, *n* (%)	3 out of 29 (10.3)	2 out of 25 (8)	1 out of 4 (25)	0.3706
CIED, *n* (%)	1 (3.2)	1 (3.8)	0 (0)	1
Clinical characteristics				
Fever, *n* (%)	30 (96.8)	26 (100)	4 (80)	0.1613
Embolic phenomena, *n* (%)	15 out of 30 (50)	10 out of 25 (40)	5 (100)	0.0421
Paravalvular abscess, *n* (%)	10 (32.3)	9 (34.6)	1 (20)	1
Sepsis, *n* (%)	9 (29)	8 (30.8)	1 (20)	1
Heart failure, *n* (%)	8 (25.8)	7 (26.9)	1 (20)	1
Immunological phenomena, *n* (%)	5 out of 29 (17.2)	3 out of 24 (12.5)	2 (40)	0.1947
Shock, *n* (%)	2 (6.5)	2 (7.7)	0 (0)	1
Treatment				
Aminoglycoside, *n* (%)	13 (41.9)	10 (38.5)	3 (60)	0.6254
Penicillin, *n* (%)	12 (38.7)	10 (38.6)	2 (40)	1
Cephalosporin, *n* (%)	12 (38.7)	8 (30.8)	4 (80)	0.060
Aminopenicillin, *n* (%)	9 (29)	8 (30.8)	1 (20)	1
Carbapenem, *n* (%)	6 (19.4)	6 (23.1)	0 (0)	0.5533
Antipseudomonal penicillin, *n* (%)	3 (9.7)	3 (11.5)	0 (0)	1
Quinolone, *n* (%)	1 (3.2)	1 (3.8)	0 (0)	1
Surgical management, *n* (%)	20 (64.5)	17 (65.4)	3 (60)	1
Outcomes				
Deaths due to infection, *n* (%)	2 (6.5)	NA	NA	NA
Deaths overall, *n* (%)	5 (16.1)	NA	NA	NA

CIED: cardiac implanted electronic device; IE: infective endocarditis; IQR: interquartile range; IVDU: intravenous drug use; NA: not applicable; *: data are among the number of patients mentioned on top unless otherwise described.

**Table 2:** Characteristics of the included studies.

Study	Number of Patients	Age (Years)	Gender	Site of Infection *n*	Microbiology of Infection, *n*	Treatment Administered, *n*	Infection Outcomes, *n*
Shankar et al., 1980 [32]	1	64	Male 1	TrV 1	Dysgonic fermenter type-2 1	Penicillin 1	Clinical cure ^a^ 0 Deaths overall 1 Deaths due to IE 1
Montejo Baranda et al., 1984 [33]	1	43	Female 1	MV 1	*C. canimorsus* 1	Penicillin 1	Clinical cure 1 Deaths overall 0
Worthington et al., 1984 [34]	1	59	Female 1	TrV 1	Dysgonic fermenter type-2 1	Cephalosporin 1 Aminoglycoside 1 Surgical management 1	Clinical cure 0 Deaths overall 1 Deaths due to IE 0
Archer et al., 1985 [35]	1	39	Male 1	MV 1	Dysgonic fermenter type-2 1	Aminopenicillin 1	Clinical cure 1 Deaths overall 0
Buu-Hoi et al., 1988 [36]	1	30	Male 1	NR 1	*C. ochracea* 1	Aminopenicillin 1	Clinical cure 1 Deaths overall 0
Niefield et al., 1988 [37]	1	47	Male 1	TrV 1	Dysgonic fermenter type-2 1	Penicillin 1 Aminoglycoside 1 Surgical management 1	Clinical cure 1 Deaths overall 0
Gormand et al., 1989 [38]	1	61	Male 1	MV 1	*C. ochracea* 1	Penicillin 1 Surgical management 1	Clinical cure 1 Deaths overall 0
Adair et al., 1991 [39]	1	63	Male 1	AoV 1	*C. ochracea* 1		Clinical cure 1 Deaths overall 0
Decoster et al., 1992 [40]	1	52	Male 1	AoV 1	*C. canimorsus* 1	Penicillin 1	Clinical cure 1 Deaths overall 0
Andersen et al., 1992 [41]	1	56	Male 1	TrV 1	*C. canimorsus* 1	Penicillin 1 Aminoglycoside 1	Clinical cure 1 Deaths overall 0
Kooter et al., 1999 [42]	1	69	Female 1	TrV 1	*C. canimorsus* 1	Penicillin 1 Cephalosporin 1 Aminoglycoside 1	Clinical cure 1 Deaths overall 0
Ngaage et al., 1999 [43]	1	63	Male 1	AoV 1	*C. canimorsus* 1	Penicillin 1 Surgical management 1	Clinical cure 1 Deaths overall 0
Frigiola et al., 2003 [44]	1	41	Female 1	MV 1	*C. canimorsus* 1	Cephalosporin 1 Surgical management 1	Clinical cure 1 Deaths overall 0
Wareham et al., 2006 [31]	1	42	Male 1	AoV 1	*C. canimorsus* 1	Penicillin 1 Cephalosporin 1 Aminoglycoside 1 Surgical management 1	Clinical cure 1 Deaths overall 0
Gutierrez-Martin et al., 2007 [45]	1	51	Male 1	AoV 1	*C. haemolytica* 1	Aminopenicillin 1 Cephalosporin 1 Aminoglycoside 1 Surgical management 1	Clinical cure 0 Deaths overall 1 Deaths due to IE 1
Mills et al., 2008 [46]	1	64	Male 1	NR 1	*Capnocytophaga* genomospecies AHN 8471	Penicillin 1 Cephalosporin 1 Aminoglycoside 1	Clinical cure 1 Deaths overall 1 Deaths due to IE 0
Hayani et al., 2009 [47]	1	55	Male 1	AoV 1 TrV 1	*C. canimorsus* 1	Carbapenem 1 Quinolone 1 Surgical management 1	Clinical cure 1 Deaths overall 0
Coutance et al., 2009 [48]	1	65	Male 1	AoV 1	*C. canimorsus* 1	Aminopenicillin 1 Aminoglycoside 1 Surgical management 1	Clinical cure 1 Deaths overall 0
Karvinen et al., 2018 [49]	1	73	Male 1	AoV 1	*C. canimorsus* 1	Aminopenicillin 1 Carbapenem 1	Clinical cure 1 Deaths overall 0
Barry et al., 2018 [50]	1	43	Male 1	AoV 1 MV 1	*C. canimorsus* 1	Cephalosporin 1 Surgical management 1	Clinical cure 0 Deaths overall 1 Deaths due to IE 0
Sakai et al., 2019 [51]	1	46	Male 1	AoV 1	*C. canimorsus* 1	Cephalosporin 1 Aminoglycoside 1 Surgical management 1	Clinical cure 1 Deaths overall 0
Cardoso et al., 2019 [52]	1	49	Female 1	TrV 1	*C. canimorsus* 1	Carbapenem 1 Surgical management 1	Clinical cure 1 Deaths overall 0
Squire et al., 2020 [53]	1	76	Female 1	CIED 1	*C. canimorsus* 1	Carbapenem 1 Surgical management 1	Clinical cure 1 Deaths overall 0
Oluyombo et al., 2021 [54]	1	70	Male 1	TrV 1	*C. canimorsus* 1	Aminopenicillin 1 Piperacillin/tazobactam 1 Cephalosporin 1 Aminoglycoside 1	Clinical cure 1 Deaths overall 0
Sri et al., 2021 [55]	1	47	Male 1	AoV 1	*C. canimorsus* 1	Aminopenicillin 1 Carbapenem 1 Aminoglycoside 1 Surgical management 1	Clinical cure 1 Deaths overall 0
Linden et al., 2021 [56]	1	70	Female 1	TrV 1	*C. canimorsus* 1	Penicillin 1 Aminopenicillin 1 Surgical management 1	Clinical cure 1 Deaths overall 0
McNicol et al., 2021 [57]	1	59	Female 1	AoV 1	*C. canimorsus* 1	Penicillin 1 Aminopenicillin 1 Aminoglycoside 1 Surgical management 1	Clinical cure 1 Deaths overall 0
Hino et al., 2022 [58]	1	60	Male 1	TrV 1	*C. canimorsus* 1	Piperacillin/tazobactam 1 Carbapenem 1 Surgical management 1	Clinical cure 1 Deaths overall 0
Harrigan et al., 2022 [59]	1	76	Male 1	AoV 1 MV 1	*C. canimorsus* 1	Cephalosporin 1 Surgical management 1	Clinical cure 1 Deaths overall 0
O’Dwyer et al., 2022 [6]	1	33	Male 1	AoV 1	*C. canimorsus* 1	Cephalosporin 1 Aminoglycoside 1 Surgical management 1	Clinical cure 1 Deaths overall 0
Rodriguez et al., 2023 [5]	1	39	Male 1	AoV 1	*C. canimorsus* 1	Piperacillin/tazobactam 1 Cephalosporin 1 Surgical management 1	Clinical cure 1 Deaths overall 0

^a^ Defined as the clinical resolution of the infection as a result of treatment. AoV: aortic valve; CIED: cardiac implantable electronic device; MV: mitral valve; PV: pulmonary valve, TrV: tricuspid valve.

## Data Availability

The data presented in this study are available on request from the corresponding author.
